# GKT137831 and hydrogen peroxide increase the release of 6-nitrodopamine from the human umbilical artery, rat-isolated right atrium, and rat-isolated vas deferens

**DOI:** 10.3389/fphar.2024.1348876

**Published:** 2024-04-05

**Authors:** José Britto-Júnior, Rafael Furlaneto, Antonio Tiago Lima, Mariana Gonçalves de Oliveira, Beatrice Severino, Francesco Frecentese, Ferdinando Fiorino, Giuseppe Caliendo, Marcelo Nicolás Muscará, Gilberto De Nucci

**Affiliations:** ^1^ Department of Pharmacology, Faculty of Medical Sciences, University of Campinas (UNICAMP), Campinas, Brazil; ^2^ Department of Pharmacy, School of Medicine, University of Naples Federico II, Naples, Italy; ^3^ Department of Pharmacology, Institute of Biomedical Sciences, University of São Paulo (USP), Sāo Paulo, Brazil; ^4^ Department of Pharmacology, Faculty of Medicine, Sao Leopoldo Mandic, Campinas, São Paulo, Brazil

**Keywords:** human umbilical vessels, nitric oxide, xanthine oxidase, dopamine, nitrocatecholamines

## Abstract

**Introduction:** The human umbilical artery (HUA), rat-isolated right atrium, and rat-isolated vas deferens present a basal release of 6-nitrodopamine (6-ND). The basal release of 6-ND from these tissues was significantly decreased (but not abolished) when the tissues were pre-incubated with N^ω^-nitro-L-arginine methyl ester (L-NAME).

**Methods:** In this study, the effect of the pharmacological modulation of the redox environment on the basal release of 6-ND was investigated. The basal release of 6-ND was measured using Liquid chromatography with tandem mass spectrometry (LC-MS/MS).

**Results and Discussion:** Pre-incubation (30 min) of the tissues with GKT137831 (1 μM) caused a significant increase in the basal release of 6-ND from all tissues. In the HUA, pre-incubation with diphenyleneiodonium (DPI) (100 μM) also caused significant increases in the basal release of 6-ND. Preincubation of the HUA with hydrogen peroxide (H_2_O_2_) (100 μM) increased 6-ND basal release, whereas pre-incubation with catalase (1,000 U/mL) significantly decreased it. Pre-incubation of the HUA with superoxide dismutase (SOD) (250 U/mL; 30 min) also significantly increased the basal release of 6-ND. Preincubation of the HUA with either allopurinol (100 μM) or uric acid (1 mM) had no effect on the basal release of 6-ND. Pre-treatment of the HUA with L-NAME (100 μM) prevented the increase in the basal release of 6-ND induced by GKT137831, diphenyleneiodonium, and H_2_O_2_. The results obtained indicate a major role of endogenous H2O2 and peroxidases as modulators of 6- ND biosynthesis/release and a lack of peroxynitrite contribution.

## Introduction

The first evidence of endogenous production of nitrocatecholamine was generated by the observation that noradrenaline levels detected by microdialysis of the rat hypothalamic paraventricular nucleus were decreased when the tissue was perfused with a solution containing nitric oxide (Shintani et al., 1996). Using 6-nitronoradrenaline synthetized by bubbling NO gas in a solution containing noradrenaline hydrochloride as a standard, extracts from the porcine brain were analyzed via electrochemical detection linked to high-pressure chromatography, and a peak with an identical retention time to the standard was identified and characterized as 6-nitronoradrenaline by UV spectrometry, mass spectrometry (MS), and Nuclear Magnetic Resonance (NMR) spectroscopy (Shintani et al., 1996). The basal release of dopamine (Britto-Júnior et al., 2020) and 6-nitrodopamine (6-ND; Britto-Júnior et al., 2021a) has been initially described from the human umbilical artery (HUA) but later identified also from other vascular tissues, such as the aortic rings of *Chelonoidis carbonarius* (Britto-Júnior et al., 2022a), *Pantherophis guttatus* (Lima et al., 2022), and *Callithrix* spp. (Britto-Júnior et al., 2023a) and in non-vascular tissues, such as rat-isolated atria (Britto-Júnior et al., 2022b) and rat- (Britto-Júnior et al., 2021b) and human-isolated vas deferens (Britto-Júnior et al., 2022c). In the cardiovascular system, 6-ND acts as a potent vasodilator and presents both positive chronotropic and inotropic effects (Zatz and De Nucci, 2023). In all the tissues mentioned above, pre-incubation of the tissues with the NO synthase (NOS) inhibitor N^ω^-nitro-L-arginine methyl ester (L-NAME) caused a significant reduction in the synthesis/release of 6-ND, indicating a major role of nitric oxide synthases in the biosynthesis of 6-ND. Indeed, the basal release of 6-ND was significantly reduced in the isolated atria of endothelial nitric oxide synthase (eNOS)^−/−^ mice but not affected when the atria were obtained from either nNOS^−/−^ or iNOS^−/−^ mice (Britto-Júnior et al., 2023b).

Acute decompensated heart failure is a complex and life-threatening clinical syndrome associated with recurrent hospitalizations and a high mortality rate (Greenberg, 2012), and the positive inotropic catecholamines, such as noradrenaline and adrenaline, can be therapeutically used to overcome end-organ hypoperfusion (Monnet et al., 2023). Although there are known factors that can precipitate acute heart failure, such as myocardial ischemia, cardiac arrhythmias, infections, and non-compliance with medication (Follath, 2009), the molecular mechanism(s) involved are not known. Since 6-ND is the most potent endogenous positive chronotropic and inotropic agent described (Britto-Júnior et al., 2023c), understanding its biosynthetic pathway may provide insights into the physiopathology of acute heart failure.

This study evaluated the effects of the pharmacological modulation of the redox environment on the production/release of 6-ND and dopamine from human umbilical arteries, rat-isolated right atrium, and rat-isolated vas deferens *in vitro*.

## Methods

### Study participants

Parturients over the age of 18, undergoing cesarean or natural delivery at the Campinas Maternity Hospital (Campinas-SP**,** Brazil) and Hospital Augusto de Oliveira Camargo (HAOC; Indaiatuba-SP, Brazil), were invited to take part in the study. The women included in this study were normotensive, did not have preeclampsia and pregestational or gestational diabetes mellitus, and none were on regular medication. Written consent was obtained from those who agreed to participate. Umbilical cords from 82 volunteers aged 18–38 years were used.

The investigation conformed to the principles outlined in the Declaration of Helsinki, and the protocol was approved by the Ethics Committee of the Institute of Biomedical Sciences of the University of São Paulo–ICB/USP (protocol number 3.165.417).

### Release of 6-ND and dopamine from human umbilical arteries (HUA)

A segment of the umbilical cord (10–20 cm) from the insertion point in the placenta and 5 cm from the umbilicus were removed by the obstetrician and placed in a container with Krebs–Henseleit solution (KHS). The Wharton’s jelly was removed, and the HUA was dissected. Two HUA rings (1.5 cm each) per subject with intact endothelium were suspended in a 3-mL organ bath containing KHS (118 mM sodium chloride, NaCl; 4.7 mM potassium chloride, KCl; 2.5 mM calcium chloride, CaCl_2_; 1.2 mM magnesium sulfate, MgSO_4_; 25 mM sodium bicarbonate, NaHCO_3_; 1.2 mM potassium phosphate monobasic, KH_2_PO_4_; and 5.6 mM glucose) with ascorbic acid (3 mM), continuously gassed with a mixture of 95% O_2_: 5% CO_2_ (pH 7.4) at 37°C for 30 min (Britto-Júnior et al., 2021a).

The isolated HUA was incubated in the absence and presence of the superoxide dismutase (SOD; 250 U/mL; 30 min), catalase (1000 U/mL; 30 min) or their combination, the non-selective free-radical scavenger resveratrol (100 μM; 30 min), the peroxynitrite scavenger uric acid (1 mM; 30 min), the glutathione peroxidase mimetic ebselen (100 μM, 30 min), and the NAD(P)H oxidase (NOX) inhibitors GSK2795039 (selective for type-2 NOX; 1 μM; 30 min) and GKT137831 (selective for type-1/4 NOX; 1 μM; 30 min) of the inhibitor of pan NOX diphenyleneiodonium (DPI, 100 μM; 30 min) and the xanthine oxidase inhibitor allopurinol (100 μM; 30 min). Another set of experiments was performed in the presence of the NO synthesis inhibitor L-NAME (100 μM) and in the presence and absence of GKT137831 (1 μM; 30 min), DPI (100 μM; 30 min), and hydrogen peroxide (H_2_O_2_) (100 μM; 30 min). An aliquot of 2 mL of the supernatant was transferred to a tube and stored at–20°C until analysis.

Generally, two HUA rings were used as controls and two HUA rings were treated in separate organ baths. The HUA rings used in each experiment were obtained from a single patient. The number of experiments is expressed as *x/y*, where *x* represents the number of parturients (umbilical cords) and *y* represents the number of samples analyzed via Liquid chromatography with tandem mass spectrometry (LC-MS/MS).

### Animals

The animals (male Wistar rats, weighing 280–320 g) were acquired from CEMIB-UNICAMP (São Paulo, Brazil). The protocols were approved by the Local Ethics Committee (CEUA; protocol no. 5746-1/2021) according to the Brazilian guidelines (CONCEA; Andersen, 2016) and the ARRIVE guidelines (Percie du Sert et al., 2020).

### Isolation of rat right atria and vas deferens

Isoflurane overdose was used for euthanasia, and the animals were exposed to a concentration greater than 5% until 1 min after breathing stopped. Exsanguination was performed to confirm the euthanasia. The right atria, right and left ventricles, and vas deferens were isolated and suspended in a 3-mL organ bath containing KHS, continuously gassed with a mixture (95% O_2_: 5% CO_2_) at 37°C and supplemented with ascorbic acid (3 mM) to prevent catecholamine oxidation. Two animals were used for each analysis, and the basal release of 6-nitrodopamine was also evaluated from tissues in the presence and absence of GKT137831 (1 μM; 30 min).

### Determination of 6-nitrodopamine and dopamine concentrations in the KHS by tandem mass spectrometry (LC-MS/MS)

The full validation of the LC-MS/MS method for the quantification of 6-ND and dopamine has been described (Campos et al., 2021; Britto-Júnior et al., 2021c). In brief, 50 μL of the internal standard (100 ng/mL of 6-nitrodopamine-d_4_ or dopamine-d_3_) was added to 1 mL of the KHS, and the samples were homogenized for 10 s. The Strata™-X 33 mm polymeric reversed SPE cartridges were pre-conditioned with 1 mL of methanol and then balanced with 2 mL of deionized water. The samples were injected into the cartridge, and the cartridge was subsequently washed three times with deionized water. The analytes were then eluted with 0.9 mL of methanol/water (90/10, v/v) with 0.1% formic acid. The eluate was evaporated under N_2_ flow at 50°C. The residue was dissolved in 100 μL of acetonitrile/water (50/50, v/v) with 0.1% formic acid and transferred to vials ready for injection. The LC–MS/MS system consisted of an LC ADVp Liquid Chromatograph Shimadzu System (Shimadzu Corporation, Kyoto, Japan) coupled to an 8060 triple quadrupole mass spectrometer (Shimadzu Corporation, Kyoto, Japan) operating in the electrospray positive ionization mode. The samples were injected into the system using an SIL-30AC autoinjector at a temperature of 8°C. The chromatography separation was performed at room temperature using a GIST-HP C_18_ column (150 mm × 3.0 mm, 3 mm) (Shimadzu, Duisburg, Germany). A 75% mobile phase A consisting of deionized water with 0.1% formic acid (v/v) and a 25% mobile phase B consisting of acetonitrile/water (90/10, v/v) with 0.1% formic acid at a flow rate of 350 μL/min were used. The injection volume was 3 µL, and the total run-time was 3.5 min. The method validation was carried out according to the United States Food and Drug Administration bioanalytical method validation guidelines (Swartz and Krull, 2003).

### Rat-isolated right atrium preparation

Euthanasia was performed by isoflurane overdose, in which animals were exposed to a concentration greater than 5% until 1 min after breathing stopped. Exsanguination was performed to confirm euthanasia. After euthanasia, the heart was removed, and the right atrium was isolated. The right atrium was mounted between two metal hooks in 10 mL custom-designed glass chambers containing the KHS, continuously gassed with a mixture of 95% O_2_: 5% CO_2_ at 37°C using a heated circulator (PolyScience, Illinois, United States of America). Tissues were allowed to equilibrate under a resting tension of 10 mN for 1 hour, and the isometric tension was registered using a PowerLab system (ADInstruments, Sydney, Australia; Britto-Júnior et al., 2022b).

### Effects of GKT137831 and hydrogen peroxide on rat-isolated atrial contraction rate

A single concentration of GKT137831 (10 μM) and H_2_O_2_ (10 μM) was added to the organ bath, and the changes in the atrial rate were monitored for 30 min. In separate experiments, the effects of GKT137831 (10 μM) and H_2_O_2_ (10 μM) were evaluated in atria pre-treated with L-NAME (100 μM; 30 min). One atrium was used for each drug and each concentration.

### Drugs and solutions

6-Nitrodopamine-d_4_ was bought from Toronto Research Chemicals Inc. (Toronto, Ontario, Canada). DPI, ebselen, GSK2795039, GKT137831, and resveratrol were purchased from Cayman Chemical Co. (Michigan, United States of America). Superoxide dismutase bovine, catalase from human erythrocytes, L-NAME, uric acid, and allopurinol were obtained from Sigma-Aldrich Chemicals Co. (St Louis, Missouri, United States of America). Dopamine-d_3_ hydrochloride was acquired from CDN Isotopes (Canada). Hydrogen peroxide was bought from Exodo Cientifica (Sumaré, São Paulo, Brazil). Strata™-X 33 mm Polymeric Reversed SPE cartridges were bought from Phenomenex (United States of America), and GIST-HP C_18_ columns were obtained from Shimadzu (Germany). Sodium chloride (NaCl), potassium chloride (KCl), calcium chloride (CaCl_2_), magnesium sulfate (MgSO_4_), sodium bicarbonate (NaHCO_3_), potassium phosphate monobasic (KH_2_PO_4_), and glucose were acquired from Merck KGaA (Darmstadt, Germany). The composition of the KHS was in mM: NaCl 118, KCl 4.7, CaCl_2_ 2.5, MgSO_4_ 1.2, NaHCO_3_ 25, KH_2_PO_4_ 1.2 and dextrose 5.6.

### Statistical analysis

The data represent the mean ± standard error of the mean (SEM). A comparison between the two groups was performed using a two-tail, unpaired Student’s t-test. *p* < 0.05 was taken as significant. The number of experiments is expressed as *x/y*, where *x* represents the number of parturients (umbilical cords) or animals and *y* represents the number of samples analyzed by LC-MS/MS. Data on the atrial rate are presented as beats per minute (bpm) before and after the respective stimulation or as the delta increase in the atrial rate.

## Results

### Effects of NOX inhibitors and NOX mimetic on the basal release of 6-ND from human umbilical arteries

**FIGURE 1:**
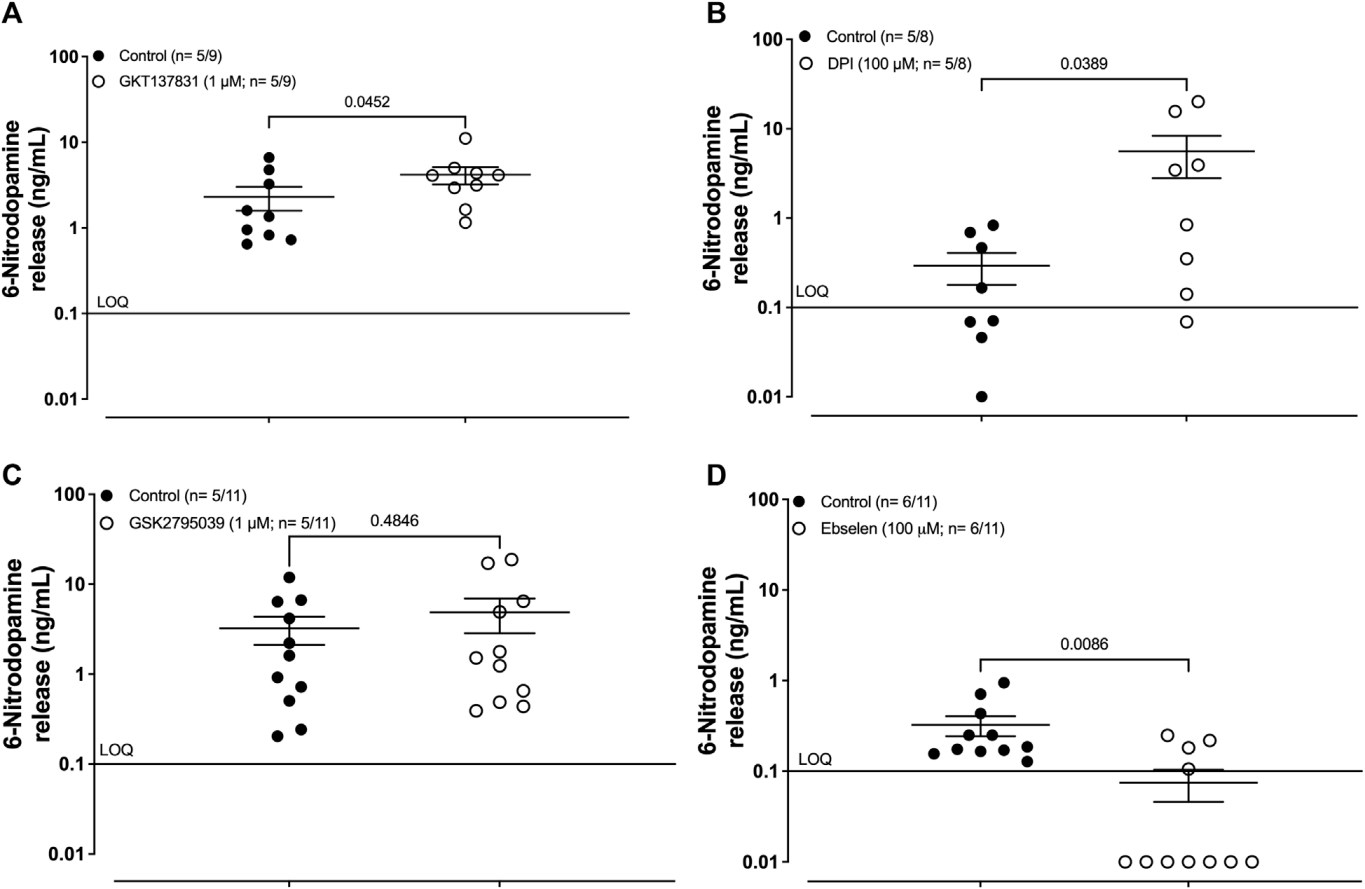
Basal release of 6-nitrodopamine in the HUA. **(A)** Effect of pre-incubation (30 min) with the selective inhibitor for type 1/4 NOX GKT137831 (1 μM), **(B)** inhibitor of pan NOX diphenyleneiodonium (DPI; 100 μM), **(C)** selective inhibitor for type 2 NOX GSK2795039, and **(D)** glutathione peroxidase-mimetic ebselen (100 μM). The number of HUAs is expressed as x/y, where x represents the number of parturients and y represents the number of samples analyzed by LC-MS/MS. LOQ, limit of quantification. Data are expressed as the mean ± SEM.

Pre-incubation of the human umbilical artery segments with the type-1/4 NOX inhibitor GKT137831 (1 μM; 30 min) caused significant increases in the basal release of 6-ND (Figure 1A; 2.30 ± 0.71 and 4.17 ± 0.96 ng/mL of control and GKT137831 1 μM, respectively; n = 5/9; *p* = 0.0452). Pre-incubation of the human umbilical artery segments with the pan NOX inhibitor diphenyleneiodonium (100 μM; 30 min) also caused significant increases in the basal release of 6-ND (Figure 1B; 0.29 ± 0.11 and 5.57 ± 2.77 ng/mL of control and DPI 100 μM, respectively; *n* = 5/8; *p* = 0.0389). Pre-incubation of the human umbilical artery segments with the type-2 NOX inhibitor GSK2795039 (1 μM; 30 min) had no effect on the basal release of 6-ND (Figure 1C; 3.23 ± 1.12 and 4.89 ± 2.04 ng/mL of control and GSK2795039 1 μM, respectively; n = 5/11; *p* = 0.4846). Pre-incubation of the human umbilical artery segments with the glutathione peroxidase mimetic ebselen (100 μM; 30 min) provoked a significant reduction in the basal release of 6-ND (Figure 1D; 0.32 ± 0.08 and 0.07 ± 0.02 ng/mL of control and ebselen 100 μM, respectively; n = 6/11; *p* = 0.0086). The results are presented in Table 1. The basal release of dopamine was not significantly altered in the analyses (Supplementary Table S1).

**TABLE 1:** Basal release of 6-nitrodopamine in the human umbilical cord artery. Effects of NOX inhibitors and NOX mimetic on the basal release of 6-ND from the HUA.

	Control (ng/mL)	Treated (ng/mL)	*p-value*	*n*
GKT137831 (1 μM)	2.3 ± 0.7	4.2 ± 0.9	0.0452	5/9
GSK2795039 (1 μM)	3.2 ± 1.1	4.9 ± 2.0	0.4846	5/11
Diphenyleneiodonium (DPI; 100 μM)	0.3 ± 0.1	5.6 ± 2.7	0.0389	5/8
Ebselen (100 μM)	0.4 ± 0.1	0.1 ± 0.1	0.0086	6/11

LOQ, limit of quantification (0.1 ng/mL).

### Effects of hydrogen peroxide, catalase, superoxide dismutase, uric acid or allopurinol on the basal release of 6-ND from human umbilical arteries

**FIGURE 2:**
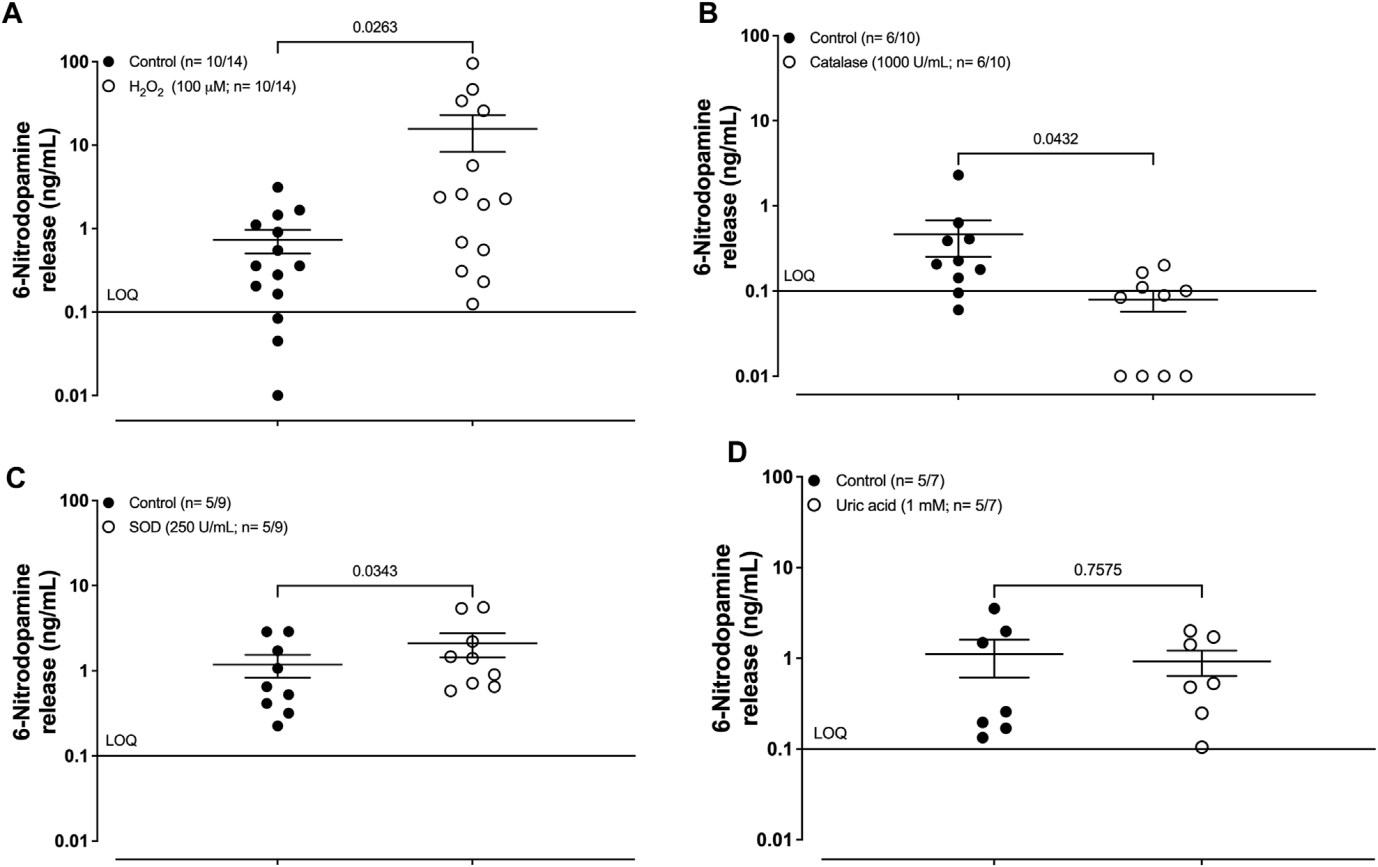
Basal release of 6-nitrodopamine in the HUA. **(A)** Effect of pre-incubation (30 min) with hydrogen peroxide (H_2_O_2_; 100 μM), **(B)** catalase (1000 U/mL), **(C)** superoxide dismutase (SOD; 250U/mL), and **(D)** uric acid (1 mM). The number of HUAs is expressed as x/y, where x represents the number of parturients and y represents the number of samples analyzed by LC-MS/MS. LOQ, limit of quantification. Data are expressed as the mean ± SEM.

Pre-incubation of the human umbilical artery segments with hydrogen peroxide (H_2_O_2_, 100 μM) caused a significant increase in the basal release of 6-ND (Figure 2A; 0.7 ± 0.2 and 15.6 ± 7.3 of control and H_2_O_2_ 100 μM, respectively; n = 10/14; *p* = 0.0263). Pre-incubation of the human umbilical artery segments with catalase (1000 U/mL; 30 min) caused a significant reduction in the basal release of 6-ND (Figure 2B; 0.5 ± 0.2 and 0.1 ± 0.1 of control and catalase 1000 U/mL, respectively; n = 6/10; *p* = 0.0432). Pre-incubation of the human umbilical artery segments with SOD (250 U/mL; 30 min) caused a significant increase in the basal release of 6-ND (Figure 2C; 1.29 ± 0.35 and 2.10 ± 0.66 ng/mL of control and SOD 250 U/mL, respectively; n = 5/9; *p* = 0.0343). Pre-incubation of the human umbilical artery segments with the peroxynitrite scavenger uric acid (1 mM; 30 min) had no effect on the basal release of 6-ND (Figure 2D; 1.1 ± 0.4 and 0.9 ± 0.3 ng/mL of control and uric acid 1 mM, respectively; *n* = 5/7; *p* = 0.7575). Pre-incubation of the human umbilical artery segments with the xanthine oxidase inhibitor allopurinol (100 μM; 30 min) had no effect on the basal release of 6-ND (Table 2). The basal release of dopamine was not significantly altered in the analyses (Supplementary Table S1).

**TABLE 2:** Basal release of 6-nitrodopamine in the human umbilical cord artery. Effects of hydrogen peroxide, catalase, superoxide dismutase, uric acid, and allopurinol on the basal release of 6-ND from the HUA.

	Control (ng/mL)	Treated (ng/mL)	*p-value*	*n*
H_2_O_2_	0.7 ± 0.2	15.6 ± 7.3	0.0263	10/14
Catalase (1000 U/mL)	0.5 ± 0.2	0.1 ± 0.1	0.0432	6/10
SOD (250 U/mL)	1.2 ± 0.3	2.1 ± 0.6	0.0343	5/9
Uric acid (1 mM)	1.1 ± 0.4	0.9 ± 0.3	0.7575	5/7
Allopurinol (100 μM)	7.1 ± 3.8	5.2 ± 1.9	0.6632	9/9

LOQ, limit of quantification (0.1 ng/mL).

### Effects of NOX inhibitors and hydrogen peroxide on release of 6-ND from L-NAME-treated HUAs

**FIGURE 3:**
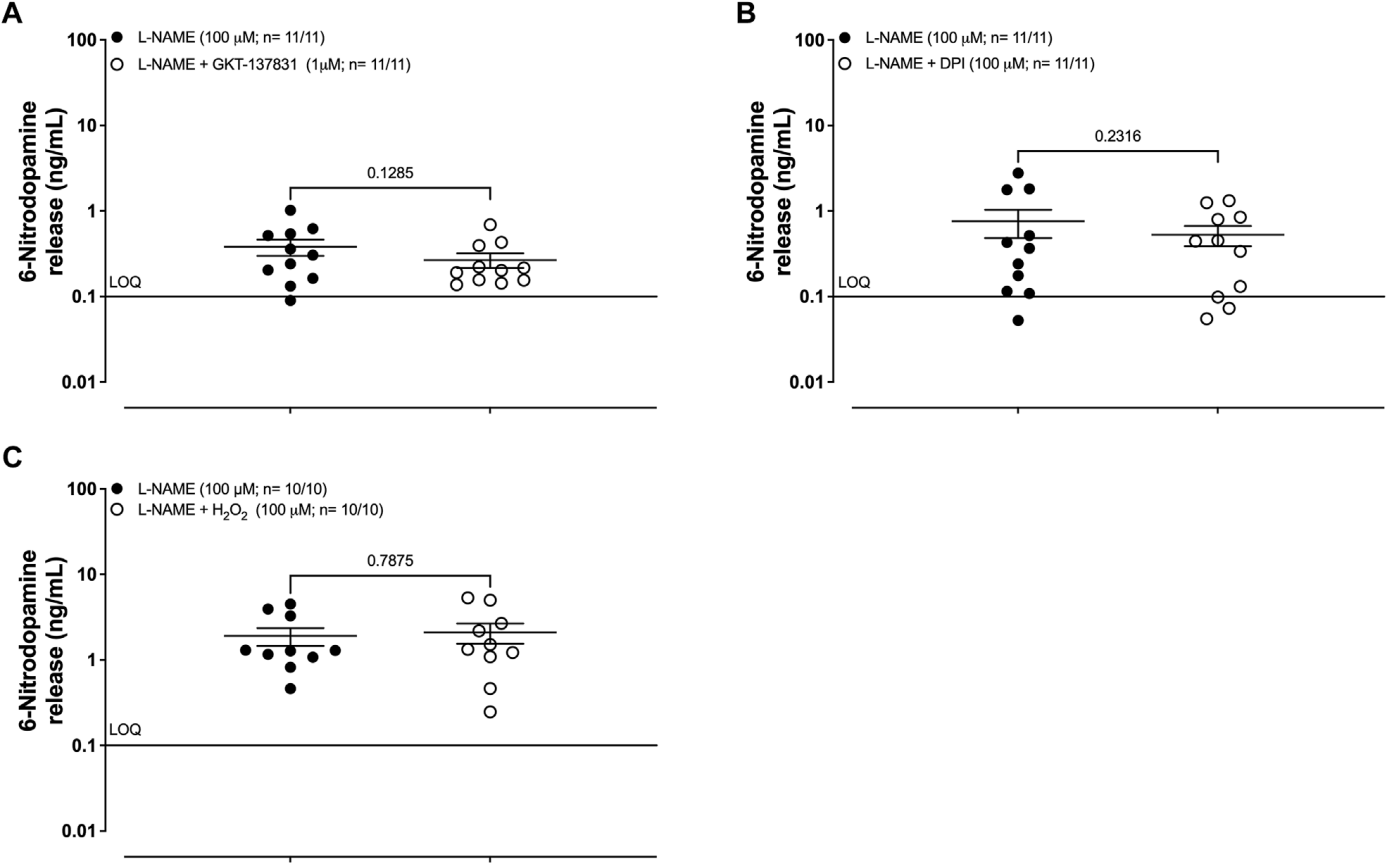
Basal release of 6-nitrodopamine in the HUA. L-NAME (100 μM; 30 min) pre-treated human umbilical artery segments. **(A)** Effect of pre-incubation (30 min) with the selective inhibitor for type 1/4 NOX GKT137831 (1 μM), **(B)** inhibitor of pan NOX diphenyleneiodonium (DPI; 100 μM), and **(C)** hydrogen peroxide (H_2_O_2_; 100 μM). The number of HUAs is expressed as x/y, where x represents the number of parturients and y represents the number of samples analyzed by LC-MS/MS. LOQ, limit of quantification. Data are expressed as the mean ± SEM.

In L-NAME (100 μM; 30 min) pre-treated human umbilical artery segments, incubation with the type-1/4 NOX inhibitor GKT137831 (1 μM; 30 min) failed to cause a significant increase in the basal release of 6-ND (Figure 3A; 0.4 ± 0.1 and 0.3 ± 0.1 ng/mL of control and treated, respectively; n = 11/11; *p* = 0.1285). Incubation of the human umbilical artery segments with L-NAME (100 μM; 30 min) also prevented the increases in the basal release of 6-ND (Figure 3B; 1.9 ± 0.5 and 2.1 ± 0.6 ng/mL of control and treated, respectively; n = 11/11; *p* = 0.2316) induced by the pan NOX inhibitor diphenyleneiodonium (100 μM; 30 min). Incubation of the human umbilical artery segments with L-NAME (100 μM; 30 min) blocked the increase in the basal release of 6-ND (Figure 3C) induced by hydrogen peroxide (H_2_O_2_, 100 μM). The results are presented in Table 3.

**TABLE 3:** Basal release of 6-nitrodopamine in the human umbilical cord artery. Effects of NOX inhibitors and hydrogen peroxide in L-NAME treated (100 μM) from the HUA.

	Control (ng/mL)	Treated (ng/mL)	*p-value*	*n*
GKT137831 (1 μM)	0.4 ± 0.1	0.3 ± 0.1	0.1285	11/11
Diphenyleneiodonium (DPI; 100 μM)	0.8 ± 0.3	0.5 ± 0.1	0.2316	11/11
H_2_O_2_ (100 μM)	1.9 ± 0.5	2.1 ± 0.6	0.7875	10/10

LOQ, limit of quantification (0.1 ng/mL).

### Effects of GKT137831 on the basal release of 6-ND from rat-isolated right atria and vas deferens

**FIGURE 4:**
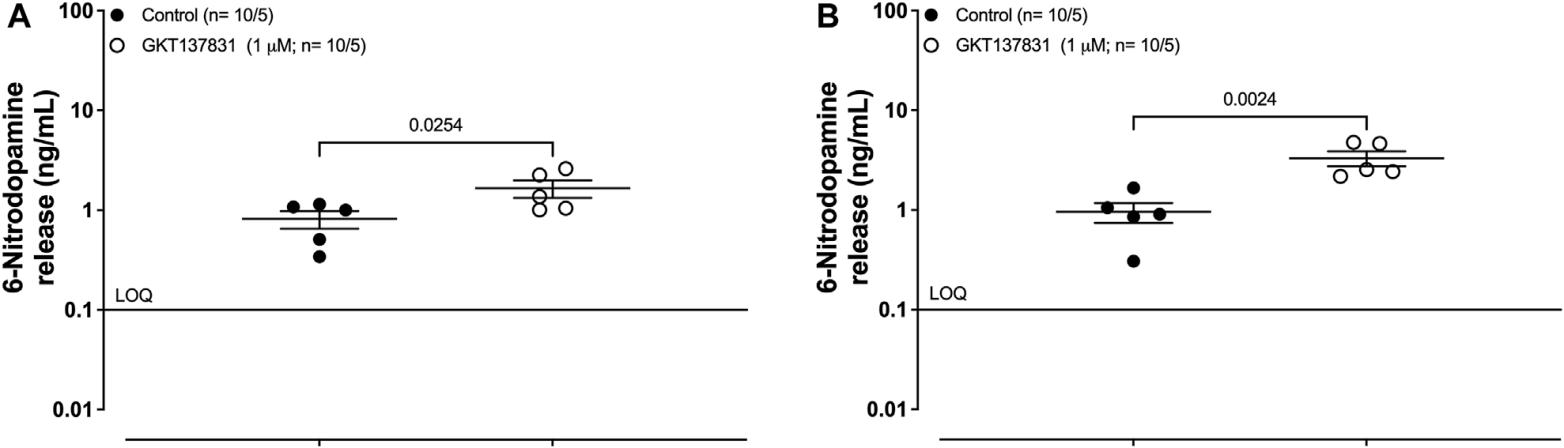
Basal release of 6-nitrodopamine in rat-isolated right atria and vas deferens. The release of 6-ND in atria **(A)** and vas deferens **(B)** was detected in KHS via LC-MS/MS. GKT137831, the selective inhibitor for type 1/4 NOX (1 μM; 30 min), causes significant increases in the basal release of 6-ND. The number of right atria and vas deferens is expressed as x/y, where *x* represents the number of animals and *y* represents the number of samples analyzed via LC-MS/MS. LOQ, limit of quantification. Data are expressed as the mean ± SEM.

Pre-incubation of the rat isolated right atrium with the type-1/4 NOX inhibitor GKT137831 (1 μM; 30 min) caused significant increases in the basal release of 6-ND (Figure 4A). Similar results were obtained with the rat-isolated vas deferens (Figure 4B). The results are presented in Table 4. In the rat-isolated right atrium, the basal release of dopamine was not altered after pre-incubation with GKT137831 (1 μM; 30 min). However, in the rat-isolated vas deferens, GKT137831 caused a significant decrease in the basal release of dopamine (Supplementary Table S2).

**TABLE 4:** Basal release of 6-nitrodopamine in rat-isolated right atria and vas deferens. Effect of GKT137831 on the basal release of rat-isolated right atria and vas deferens.

	Control (ng/mL)	Treated (ng/mL)	*p-value*	*n*
Right atria	1.0 ± 0.2	3.3 ± 0.6	0.0254	10/5
Vas deferens	0.8 ± 0.2	1.7 ± 0.3	0.0024	10/5

LOQ, limit of quantification (0.1 ng/mL).

### Effects of GKT137831 and hydrogen peroxide on the rat-isolated right atria rate

**FIGURE 5:**
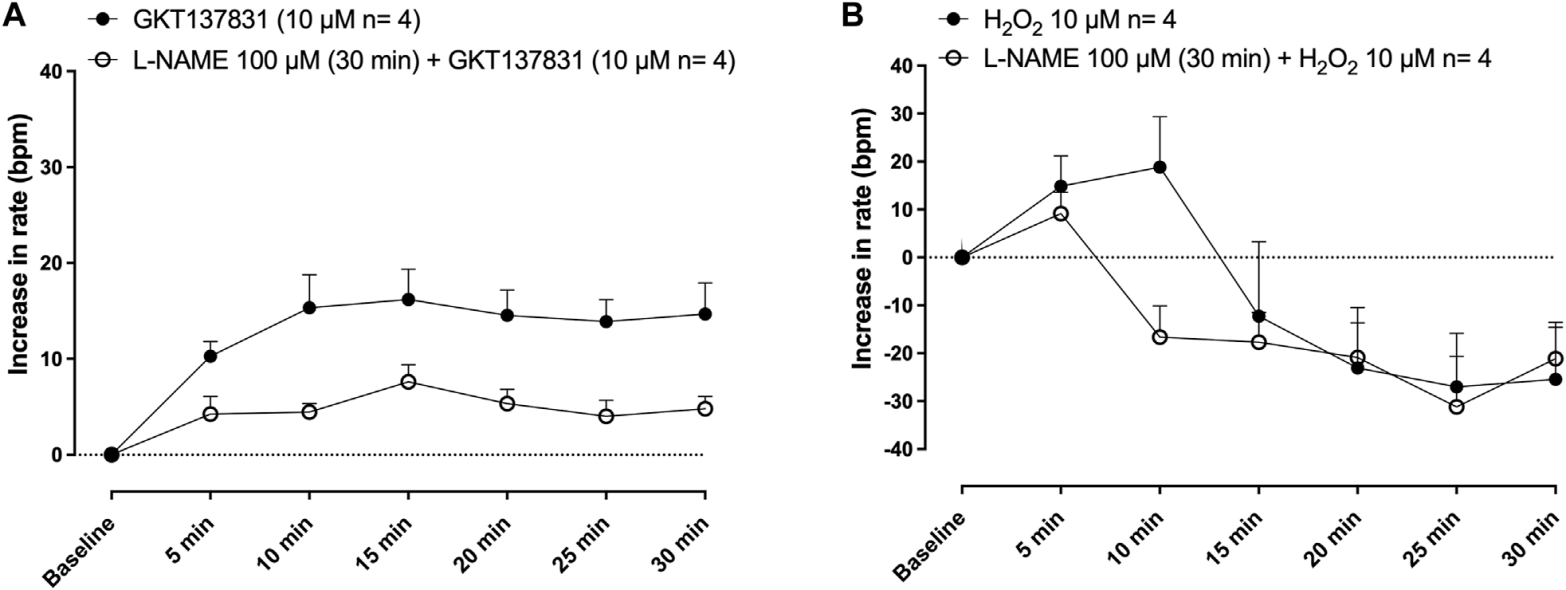
Effect of GKT137831 and hydrogen peroxide on the rat-isolated right atria rate. Selective inhibitors for type 1/4 NOX, GKT137831 (10 μM) **(A)** and H_2_O_2_ (10 μM 30 min) **(B)** caused increases in the frequency of the rat-isolated right atrium and were attenuated with pre-treatment with L-NAME (100 μM, 30 min).

The selective inhibitor for type 1/4 NOX GKT137831 (10 μM 30 min; Figure 5A; *n* = 4) and H_2_O_2_ (10 μM 30 min; Figure 5B; *n* = 4) caused increases in the frequency of the rat isolated right atrium. The pre-treatment with L-NAME (100 μM, 30 min) attenuated the increases induced by either GKT137831 (Figure 5A) or H_2_O_2_ (Figure 5B) increase in the frequency of the rat isolated right atria.

## Discussion

The results obtained indicate a potential major role of endogenous hydrogen peroxide and peroxidases in the biosynthesis of 6-ND in human umbilical cord vessels. Hydrogen peroxide is an end product of several metabolic reactions by membrane-associated NADPH oxidases (NOX) and can also be generated as a by-product of mitochondrial respiration (Winterbourn, 2020). Hydrogen peroxide is produced by endothelial cells; in experiments performed with small mesenteric arteries from mice, we loaded the arteries with dichlorodihydrofluorescein diacetate (DCF), a peroxide-sensitive fluorescence dye (Ohba et al., 1994), and observed them using a laser confocal microscope. Stimulation with acetylcholine caused a significant increase in fluorescence in the endothelium, which was virtually abolished by pre-incubation of the endothelial cells with catalase (Matoba et al., 2000). Hydrogen peroxide can cross membranes, facilitated by aquaporins (Erudaitius et al., 2017; Bestetti et al., 2020). Hydrogen peroxide is rapidly metabolized by peroxiredoxins, catalase, and GSH peroxidase (Winterbourn, 2020) and can also react with CO_2_, forming peroxymonocarbonate (HCO_4_^−^). The significant reduction in the basal release of 6-ND obtained with catalase supports the importance of hydrogen peroxide in 6-ND biosynthesis. The peroxidase mimetic ebselen consumes hydrogen peroxide for the oxidation of reduced glutathione (Sies, 1993). Indeed, peroxiredoxins and glutathione peroxidases are abundant throughout the cells and are very efficient in metabolizing H_2_O_2_, with constant rates in the 10^6^–10^8^ M^-1^ s^-1^ range (Winterbourn, 2020). However, an alternative mechanism by which ebselen decreases the basal release of 6-ND could be due to its ability to inhibit eNOS (Zembowicz et al., 1993).

Rat skeletal muscle cell cultures produce hydrogen peroxide, and intense electrical stimulation increases H_2_O_2_ formation (Silveira et al., 2003). Exposure of rat cardiomyocytes to H_2_O_2_ causes gradual increases in intracellular calcium (Wang et al., 1999). In rat-isolated perfused beating atria, the average concentration of H_2_O_2_ in perfusate during the low stimulation period was 3.23 ± 0.55 nM and that during the high stimulation period was 7.00 ± 0.62 nM (Gao et al., 2011). In the isolated working heart preparation, an infusion of hydrogen peroxide (6 and 60 μM) caused a concentration-dependent increase in the heart rate (Shattock et al., 1982). Although H_2_O_2_ plays a role in heart chronotropism, further investigation is needed to determine whether the increase in the atrial rate induced by H_2_O_2_ reported here is due to either an increase in the basal release of 6-ND or increased [Ca^+2^]_i_. Although the increase was sensitive to NO inhibition, the finding that it was transient is surprising since the positive chronotropic effect of 6-ND in rat isolated atria is prolonged even after the preparation is washed (Britto-Júnior et al., 2022b).

Superoxide anion (O_2_^−^) is generated by the action of both NADH (Turrens and Boveris, 1980) and NADPH oxidases and xanthine oxidases. The superoxide anion reacts with nitric oxide, generating peroxynitrite (Blough and Zafiriou, 1985; Beckman and Koppenol, 1996). Although the main pathway for O_2_^-^ elimination in biological systems is SOD-catalyzed dismutation to H_2_O_2_ and O_2_, NO is the only biomolecule known to react fast enough and to be produced at sufficient concentrations to outcompete SOD for its reaction with O_2_^-^, yielding peroxynitrite. The nitration induced by peroxynitrite could be due to several different mechanisms: the formation of the nitronium cation (NO^+^) or the formation of the nitrogen dioxide radical (^**•**^NO_2_). The formation of the nitronium cation is favored over the formation of nitrogen dioxide radical in the presence of free metal ions. Another mechanism by which peroxynitrite can generate the nitrogen dioxide radical (^**•**^NO_2_) is through the reaction with CO_2_, yielding nitrosoperoxocarboxylate (ONOOCO_2_^−^) that rapidly decays into carbonate radical (^**•**^CO_3_) and nitrogen dioxide radical (^**•**^NO_2_), promoting one-electron oxidations and nitrations (Radi, 2022). However, our results indicate that peroxynitrite formation is not involved in the biosynthesis of 6-nitrodopamine since the synthesis/release of 6-ND is augmented with pre-incubation with SOD. Uric acid has been associated with decreased NO release by cultured endothelial cells, and it increases intracellular superoxide formation (Papežíkova et al., 2013). Interestingly, uric acid is also considered a radical scavenger and antioxidant (Becker et al., 1991). However, since uric acid did not affect the release of 6-ND from the umbilical artery, it indicates that these actions of uric acid may not occur in the human umbilical artery. It is interesting that in the presence of an NO donor, in contrast to wild-type NADPH oxidase 4 (NOX4), only superoxide-producing NOX4 mutants generated peroxynitrite (Takac et al., 2011). Peroxynitrite is known to be highly reactive with thiols (Radi, 2018), forming complexes with hemoglobin, methionine, and tyrosine (Bartesaghi and Radi, 2018). Whether nitration of tyrosine may result in the synthesis of nitrocatecholamines is yet to be demonstrated.

NADPH oxidase (NOX) enzymes were first identified in neutrophils and macrophages (Sylvester et al., 2022); during phagocytosis, NOX activation produces O_2_^−^ that causes pathogen killing. In contrast to other oxidases, NADPH oxidases (NOX) produce superoxide anion as their primary and sole function (Altenhöfer et al., 2015). NOX1 is particularly abundant in the colonic epithelium and vascular smooth muscle cells (Szanto et al., 2005), whereas NOX2 is mainly found in phagocytes (Moghadam et al., 2021). NOX4 is expressed in the vascular wall and endothelial cells (Konior et al., 2014) and NOX5, although absent in rodents, is expressed in human vascular tissue (Touyz et al., 2019). GKT137831 belongs to the structural class of pyrazolopyridine-diones, acts as a preferential direct inhibitor of NOX1 and NOX4 (Aoyama et al., 2012), and does not inhibit NOX production by xanthine oxidase (Sedeek et al., 2010). The results presented herein indicate that the inhibition of NOX4 increases 6-ND biosynthesis, and this increase is dependent on the NO synthase pathway. In contrast, DPI presented a similar potency for all isoforms of NOX, and it also inhibited xanthine oxidase, acting as a general inhibitor of flavoproteins. GSK2795039 is a direct inhibitor of NOX2 and does not inhibit protein kinase C (PKC) or xanthine oxidase (Hirano et al., 2015). Our finding that 6-ND was increased with GKT137831 and the pan-inhibitor DPI presents another piece of evidence that the mechanism responsible for 6-ND synthesis does not require O_2_^−^-induced peroxynitrite production. The failure of GSK2795039 to effect 6-ND synthesis/release was expected, as NOX2 is not expressed in vascular tissues.

The identity of the reactive oxygen products originated by NOX4 is controversial. Mouse vascular smooth muscle cells (Ellmark et al., 2005), rat aortic endothelial cells (Ago et al., 2004), and the membrane fraction of the human embryonic kidney-derived (HEK) 293 cells expressing NOX (Shiose et al., 2001) exhibit NADH- and NADPH-dependent superoxide-producing activities, whereas in cell lines expressing NOX4 upon tetracycline, the superoxide anion generation was almost undetectable, although hydrogen peroxide was produced (Serrander et al., 2007). This discrepancy could be attributed to the proposed perinuclear vesicle membrane location of NOX4 (von Löhneysen et al., 2008). Therefore, the superoxide production is confined to the intravesicular space, whereas hydrogen peroxide is freely diffusible and can be measured in the extracellular medium (Werner, 2003). In a membrane-free, partially purified preparation of NOX4, 90% of the electron flux through isolated NOX4 produced H_2_O_2_, and 10% formed superoxide (Nisimoto et al., 2014). Indeed, another source of endoplasmic reticulum (ER)-derived reactive oxygen species is NOX4, which releases O_2_^−^ and H_2_O_2_ into the ER lumen to maintain its oxidizing environment (Lee et al., 2020). The kinetic mechanism of H_2_O_2_ formation is consistent with a mechanism involving the binding of one oxygen molecule, which is then sequentially reduced by the heme in two one-electron reduction steps, first to form a bound superoxide intermediate and then H_2_O_2_. Thus, it is possible that NO could react with the proposed bound superoxide intermediate. There are many sources for H_2_O_2_ production in endothelial cells (Cai, 2005). Indeed, more than 40 O_2_^−^/H_2_O_2_-generating enzymes have been identified in humans (Sies and Jones, 2020). Thus, the mechanism by which NOX inhibition increases 6-ND biosynthesis/release should be related to the inhibition of superoxide anion production.

Since the O_2_^−^/peroxynitrite pathway has been thoroughly excluded, what is the mechanism responsible for 6-ND biosynthesis? One interesting possibility is the reaction of nitrite with hydrogen peroxide, generating the nitrogen dioxide radical (^**•**^NO_2_). Hemoglobin oxidation in the erythrocyte lysate by submillimolar levels of NO_2_ only occurs in the presence of high concentrations of H_2_O_2_ (Titov and Petrenko, 2005), and this process is slowed in the presence of catalase. The nitrite-dependent oxidation of oxyhemoglobin is not affected by incubation with superoxide dismutase, indicating that, similar to our results, superoxide anion does not play an important role (Keszler et al., 2008).

The increase in the basal release of 6-ND by hydrogen peroxide is dependent on NO production since it was inhibited by L-NAME. Hydrogen peroxide induces endothelium-dependent and endothelium-independent vasorelaxation (Qiao et al., 2014), and the exposure of large-sized arteries to hydrogen peroxide causes the activation of eNOS (Tejero et al., 2019). In bovine aortic endothelial cells, hydrogen peroxide caused a potent concentration-dependent increase in NO release, as detected by the NO-specific microelectrode (Cai et al., 2003). The release was due to eNOS phosphorylation induced by phosphoinositide 3-kinase (PI 3-kinase) since it was inhibited by pre-incubation of the endothelial cells with the PI-3 kinase inhibitors wortmannin (Arcaro and Wymann, 1993) and LY294002 (Vlahos et al., 1994) and by c-Src family tyrosine kinase (Zhao et al., 1992) since it was inhibited by Src inhibitor PP1 (Karni et al., 2003). Whether these biochemical pathways are involved in 6-ND biosynthesis/release by the human umbilical artery and rat atria is under current investigation. Although solid evidence for animal NOS-like enzymes in plants is lacking (Thomas et al., 2002), few alga species possess them (Santolini et al., 2016). It is interesting that hydrogen peroxide induction of NO production in the plant *Vicia faba* could be due to an NOS-like activity since it was inhibited by L-NAME (She et al., 2004). In plants, it is assumed that the increased NO production induced by hydrogen peroxide is via the activation of nitrate reductase (Lin et al., 2012). However, in *Arabidopsis* leaves, ultraviolet B triggered significant increases in hydrogen peroxide and NO levels were regulated by GPA1, the Gα-subunit of heterotrimeric G proteins (He et al., 2004). It is interesting that in co-cultures of calf pulmonary artery endothelial cells and rabbit pulmonary artery smooth muscle cells, incubation with H_2_O_2_ was associated with a decrease in cGMP accumulation (Marczin et al., 1992). Although these results indicated that H_2_O_2_ was reducing NO levels, one more plausible explanation is that H_2_O_2_ is increasing the synthesis of 6-ND. In human-washed platelets, 6-ND does not induce an increase in either cGMP or cAMP (Nash et al., 2022).

Molybdenum-containing enzymes of the xanthine oxidase family catalyze oxygen atom transfer reactions, and nitrite can be reduced to NO with xanthine oxidase (Maia, 2023). As mentioned before, the inhibition of NO synthase causes significant reductions in the basal release of 6-ND, but it does not abolish it. However, this pathway is unlikely to be involved in 6-ND biosynthesis since the xanthine oxidase inhibitor allopurinol (Pacher et al., 2006) failed to affect the basal release of 6-ND. However, the concept of “non-dedicated nitrite reductases” (Maia and Moura, 2018) as an alternative pathway for the NOS-independent synthesis of 6-ND deserves attention. It is interesting that the basal release of 6-ND and 6-nitroadrenaline from rabbit-isolated hearts is not affected by pre-treatment of the heart with L-NAME (Junior et al., 2023). One possible pathway for the nitrosation of dopamine, independent of NOS activity, would be the reduction of endogenous nitrite/nitrate since these ions are not only the products of the metabolism of NO, but they can also act as a reservoir (Shiva, 2013). Enzymes such as hemoglobin, myoglobin, xanthine oxidoreductase (Millar et al., 1998), and cytochrome P450 reductase (Li et al., 2006) can catalyze the reduction of nitrite or nitrate to generate NO. Peroxidases are iron-containing enzymes that can catalyze one- and two-electron oxidation reactions of small anionic molecules as electron donors, such as halides, thiocyanate, and nitrite, with hydrogen peroxide (García et al., 2023). Nitrite (NO_2_^−^) is a symmetrical anion and, therefore, can undergo either oxidation or reduction, generating nitrogen dioxide (^**•**^NO_2_) or nitric oxide, respectively.

Diphenyleneiodonium is also considered a potent and irreversible inhibitor of nitric oxide synthase (Rand and Li, 1993; Szilagyi et al., 2016), yet it promoted a remarkable increase in the 6-ND synthesis/release from the umbilical artery, and this increase was sensitive to NOS inhibition since it was not observed in the L-NAME-treated umbilical artery. How should this apparent paradox be explained? The most likely explanation is that DPI either does not inhibit NOS in the human umbilical artery, or else it causes a transient inhibition of NOS. Indeed, DPI produces only a transient pressor response following systemic administration to animals, and the dose-dependent increases in the mean arterial pressure induced by L-NAME in conscious rats were shifted to the right by pre-treatment with DPI (Wang et al., 1995). Since 6-ND is a potent vasodilator (Zatz and De Nucci, 2023), it is possible that an increase in the basal release of 6-ND induced by DPI counteracts the vasoconstriction induced by the lack of NO *in vivo*.

However, how does DPI increase the release of 6-ND in the umbilical artery? Mammalian cells and tissues actively decompose NO (Gardner, 2005). NO dioxygenases (NODs) convert NO to nitrate; they bind O_2_ to form a stable heme-Fe^3+^(O_2_^−^) complex that reacts rapidly with NO, forming nitrate, and they are inhibited by DPI (Gardner et al., 2001). One possibility is that the inhibition of NOX by DPI could explain both the remarkable increase in 6-ND and the sensitivity to NOS inhibition. Another possibility would be that the inhibition of superoxide anion production by DPI plays a major role in 6-ND biosynthesis.

GKT137831 enhances the basal release of 6-ND from both rat-isolated atria and vas deferens. Since in the former tissue, eNOS is the main isoform responsible for 6-ND synthesis (Britto-Júnior et al., 2023b), whereas in the latter tissue, nNOS is the main isoform associated with 6-ND release (Britto-Júnior et al., 2024), one should consider that this mechanism of action could be relevant for the potential therapeutic effects of GKT137831. For instance, GKT137831 improves erectile function in diabetic rats (Zhao et al., 2015), and one possible explanation could be that GKT137831 induces increases in 6-ND in the corpus cavernosum. Although *in vivo* administration of GKT137831 induces dose-dependent reno- and atheroprotection in established micro- and macrovascular diseases (Gray et al., 2017), this is apparently the first report of a positive chronotropic effect of this NOX1/4 inhibitor. Since this effect was significantly reduced by pre-treatment of the atria with L-NAME, it is possible that the mechanism of action is related to the increase in 6-ND biosynthesis/release.

**FIGURE 6:**
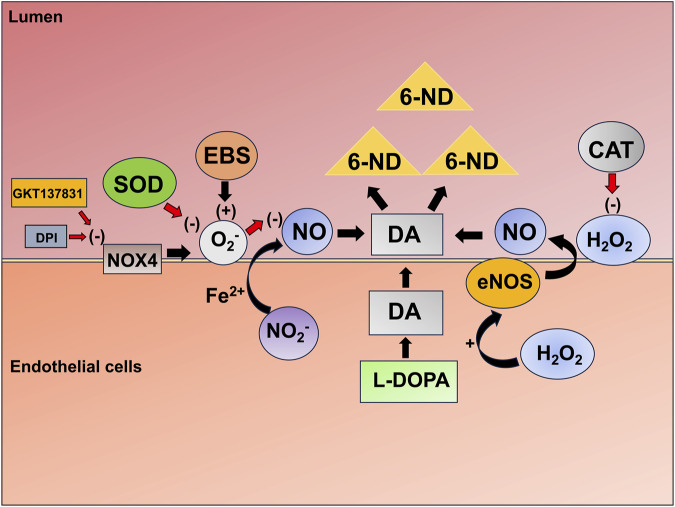
Proposed mechanisms involved in 6-ND biosynthesis/release. Endothelial nitric oxide synthase releases NO that is important for the “nitration” of dopamine. Hydrogen peroxide stimulates eNOS, therefore increasing the biosynthesis/release of 6-ND. Catalase destroys hydrogen peroxide; therefore, it causes a reduction in the biosynthesis/release of 6-ND. NOX4 produces a superoxide anion (O_2_^−^), which destroys nitric oxide and therefore reduces the biosynthesis/release of 6-ND. The NOX4 inhibitor GKT137831 and the NOX pan-inhibitor diphenyleneiodonium cause increases in the biosynthesis/release of 6-ND by inhibiting the mechanism mentioned above, and the peroxidase mimetic ebselen (EBS) decreases the biosynthesis/release of 6-ND by generating O_2_^−^. Nitrite (NO_2_^-^) can be reduced to NO by *facultative* nitrite reductases in the presence of Fe^2+^ as an NOS-independent pathway.

The increase in 6-ND levels induced by the NOX1/4 inhibitor in the rat-isolated vas deferens was accompanied by a concomitant decrease in dopamine levels. However, the increase in 6-ND levels (0.9 ng/mL) was significantly higher than the observed decrease in dopamine levels (0.3 ng/mL). In the rat atria, the increase in 6-ND levels was not accompanied by changes in dopamine levels. As proposed above, the main mechanism responsible for the increase in 6-ND levels by GKT137831 could be the inhibition of superoxide anion production, and this mechanism could also be responsible for an increase in dopamine availability. Dopamine is a very good antioxidant (Iuga et al., 2011), and although the superoxide anion is not a very reactive species, the hydroperoxyl radical (•OOH), which is the protonated form of the superoxide anion (DeGrey, 2002), presents higher reactivity and could contribute significantly to dopamine oxidation. The possible mechanisms involved in the synthesis/release of 6-ND are depicted in Figure 6.

## Conclusion

The results indicate that hydrogen peroxide and peroxidases, rather than peroxynitrite, play a major role in the biosynthesis/release of 6-ND.

## Data Availability

The original contributions presented in the study are included in the article/Supplementary Material; further inquiries can be directed to the corresponding author.
