# Supplementary material for: GKT137831 and hydrogen peroxide increase the release of 6-nitrodopamine from the human umbilical artery, rat-isolated right atrium, and rat-isolated vas deferens
Source: Front Pharmacol. 2024 Apr 5;15:1348876. doi: 10.3389/fphar.2024.1348876 (PMC11026650; doi:10.3389/fphar.2024.1348876)
Supplement: Supplementary file 1 [file Table1.docx]

Table S1. Basal release of dopamine in human umbilical cord artery (HUA)

|  | Control (ng/mL) | Treated (ng/mL) | *P-value* | *n* |
| --- | --- | --- | --- | --- |
| GKT137831 (1 μM) | 0.2 ± 0.1 | 0.3 ± 0.1 | 0.5771 | 5/9 |
| GSK2795039 (1 μM) | 0.8 ± 0.5 | 2.1 ± 1.3 | 0.3796 | 5/11 |
| Diphenyleneiodonium (DPI; 100 μM) | 0.1 ± 0.1 | 0.2 ± 0.1 | 0.1627 | 5/6 |
| Ebselen (100 μM) | 1.0 ± 0.2 | 0.7 ± 0.1 | 0.1897 | 5/11 |
| H_2_O_2_ | 3.2 ± 1.1 | 7.9 ± 4.7 | 0.1737 | 10/14 |
| Catalase (1000 U/mL) | 0.4 ± 0.1 | 0.3 ± 0.1 | 0.1785 | 6/10 |
| SOD (250 U/mL) | 0.2 ± 0.1 | 0.4 ± 0.2 | 0.4163 | 5/9 |
| Uric acid (1 mM) | 0.1 ± 0.1 | 0.2 ± 0.1 | 0.6478 | 5/7 |

LOQ: limit of quantification (0.1 ng/mL).

Table S2. Basal release of dopamine (DA) in rat isolated right atria and vas deferens. Effect of GKT137831 on the basal release of DA in rat isolated right atria and vas deferens

|  | Control (ng/mL) | Treated (ng/mL) | *P-value* | *n* |
| --- | --- | --- | --- | --- |
| Right atria | 0.5 ± 0.1 | 0.5 ± 0.2 | 0.4151 | 10/5 |
| Vas deferens | 0.8 ± 0.1 | 0.5 ±0.1 | 0.0059 | 10/5 |

LOQ: limit of quantification (0.1 ng/mL).
